# Designing and Immunomodulating Multiresponsive Nanomaterial for Cancer Theranostics

**DOI:** 10.3389/fchem.2020.631351

**Published:** 2021-01-29

**Authors:** Amreen Khan, Faith Dias, Suditi Neekhra, Barkha Singh, Rohit Srivastava

**Affiliations:** ^1^ Department of Biosciences and Bioengineering, Indian Institute of Technology Bombay, Mumbai, India; ^2^ Centre for Research in Nanotechnology and Science, Indian Institute of Technology Bombay, Mumbai, India; ^3^ Department of Chemical Engineering, Thadomal Shahani Engineering College, Mumbai, India

**Keywords:** immunomodulation, nanomedicine, cancer, nanomaterials, theranostics, imaging, clinical trials

## Abstract

Cancer has been widely investigated yet limited in its manifestation. Cancer treatment holds innovative and futuristic strategies considering high disease heterogeneity. Chemotherapy, radiotherapy and surgery are the most explored pillars; however optimal therapeutic window and patient compliance recruit constraints. Recently evolved immunotherapy demonstrates a vital role of the host immune system to prevent metastasis recurrence, still undesirable clinical response and autoimmune adverse effects remain unresolved. Overcoming these challenges, tunable biomaterials could effectively control the co-delivery of anticancer drugs and immunomodulators. Current status demands a potentially new approach for minimally invasive, synergistic, and combinatorial nano-biomaterial assisted targeted immune-based treatment including therapeutics, diagnosis and imaging. This review discusses the latest findings of engineering biomaterial with immunomodulating properties and implementing novel developments in designing versatile nanosystems for cancer theranostics. We explore the functionalization of nanoparticle for delivering antitumor therapeutic and diagnostic agents promoting immune response. Through understanding the efficacy of delivery system, we have enlightened the applicability of nanomaterials as immunomodulatory nanomedicine further advancing to preclinical and clinical trials. Future and present ongoing improvements in engineering biomaterial could result in generating better insight to deal with cancer through easily accessible immunological interventions.

## Introduction

**FIGURE 1:**
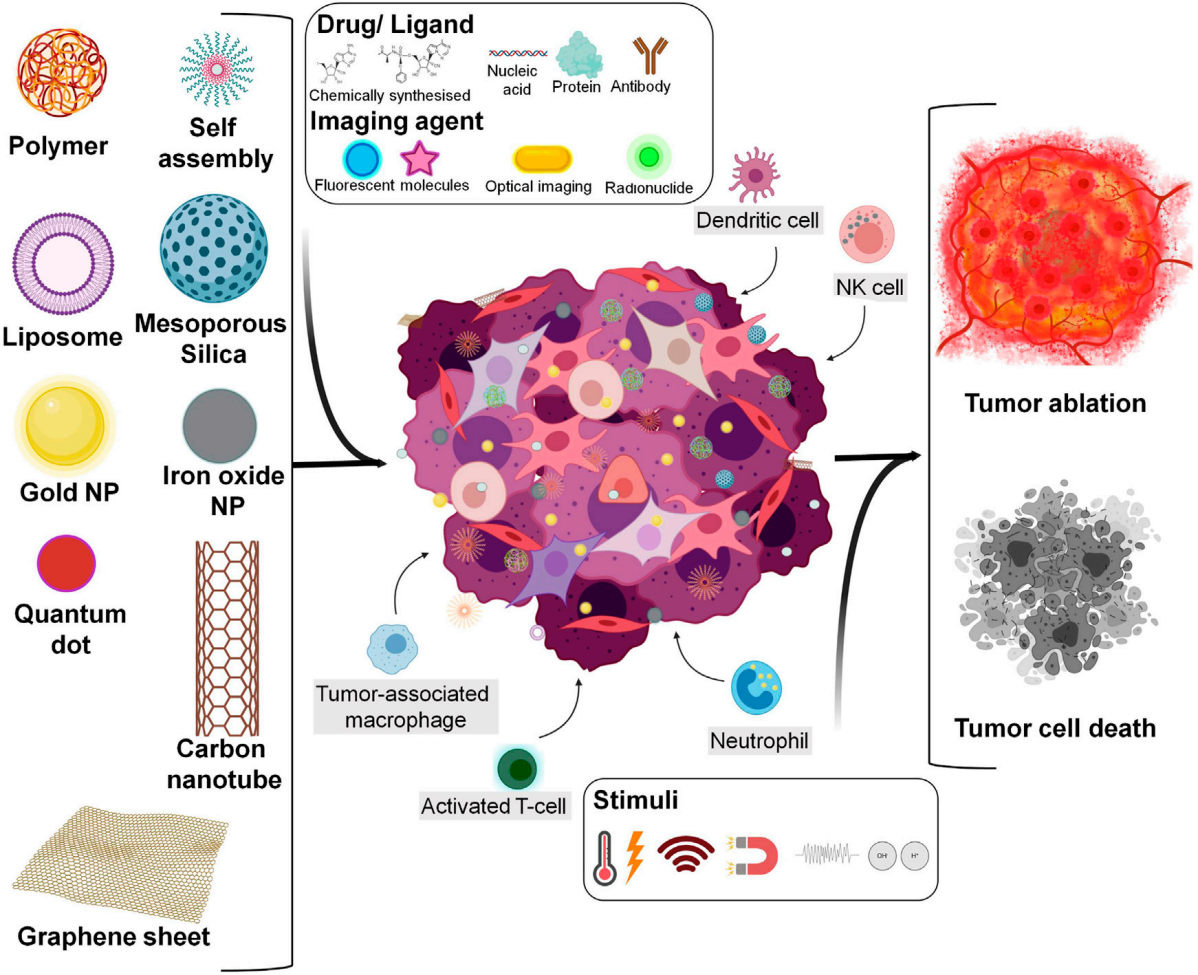
Overview stating types of engineered nanomaterial synchronised with immune cell activation for tumor destruction (Created with BioRender.com).

Cancer is a notable disease indexing high mortality rate worldwide as statistics had estimated approximately 18 million emerging cases and 9.6 million deaths during 2018 (Bray et al., 2018; Siegel et al., 2020). Amongst which Asian countries, lung cancer in male and breast cancer in female leads the chart with several folds. Surveillance data has reported to be limited in the countries that state epidemic at its early stage. Poor prognoses, diagnosis, and treatment in the view of variation amongst population could be relatable reason for such restrictions (Bray et al., 2018). Trend forecasting intrinsic and extrinsic factors are causative of fluctuation in cancer demography. Likewise, age-based cancer analysis has stipulated to reveal 89,500 new cases and 9,270 deaths in United States alone during the year 2020 with individuals between 15 and 39 years (Miller et al., 2020). Program such as Cancer Moonshot 2020 further proposes to awaken a diverse research community to keep working towards more potentially driven therapeutic strategies (Wang, 2016). Ahead of this, marked as inexplicable due to difficulty in diagnosis and recurrence, developing effective strategies to deal with cancer is in enormous demand. Individual conventional therapies such as chemo, radiation, and surgical methods are not sufficient to fully cure cancer. Another concept as immunotherapy evolved through assessing defensive behavioral responses resulting from the natural process of immune system displayed when they are on the verge to encounter malicious tumors by activation, modulation, suppression, etc. activities (Song et al., 2017). Coordination of different immune cells including Dendritic cells (DCs), NK cells, T-cells and macrophages results to induce variable reposes. Wherein the NK cells release porphyrin and granzyme toxin collectively into the target cells enhancing tumor apoptosis. T-cells are primary contributors of adaptive immune system. They are grouped into subsets according to the cluster of differentiation molecules expressed and role in immune system. Like T-cells, macrophages are also classified based on the functions they perform. M1 macrophages activate to secrete some pro-inflammatory cytokines participating in tumor cell destruction. In contrast, M2 macrophages work to produce anti-inflammatory cytokine combating the inflammatory response. DCs function mainly in processing and presenting antigen to T-cells facilitating antitumor immunity (Chulpanova et al., 2020). Collectively, immunotherapy for cancer is upsurging as the golden key by regulating the immune system and unraveling difficulties of personalized medicine. With the enhancement of immune strength being considered as rational of immunotherapy, immunomodulatory cancer theranostics aims to achieve the unmet clinical needs for providing effective antitumor therapy protruding minimum off-target effects in a reliable way covering a wide range of heterogeneous cells (Nam et al., 2019). One way to classify immunomodulation is based on the response they tend to produce. Specific modulation uses molecules like adjuvants directed towards activating target of interest, especially B-cells and T-cells but might result in ineffective mobilization. Whereas, the non-specific molecules like cytokines act as a depot for sustained release to boost the immune system and address symptomatic issues when the underlying cause is unclear. At times, such type of immunomodulation could be more potent often generating a better subtherapeutic effect than specific modulations but provoke severe immunotoxicity (Fang and Zhang, 2016). Apart from primary cells, discrete components of the immune system like mediators, checkpoints, tumor-associated macrophages (TAMs), Tumor-associated fibroblasts (TAFs), toll-like receptors (TLRs), myeloid-derived suppressor cells (MDSCs), nucleotide-binding oligomerization domain (NOD)-like receptors (NLRs), retinoic acid-inducible gene I (RIG-I)-like receptors (RLRs), and C-type lectins, etc. could also be utilized for immunomodulation (Song et al., 2017; Le et al., 2019). On the other side, immune response and related activities could also be supported by well-constructed nanobiomaterial having good carrier capacity. Scheme of tumor destruction by different nanomaterials and their modifications synergized with immune cell recruitment has been illustrated in Figure 1. A more comprehensive correlation of theranostic nanomaterial and immunomodulation will be discussed in the following sections.

Nanomaterial is a widely researched domain to transform various aspects of technology. Theranostics, an integration of therapeutically active treatment and diagnosis of disease are an important application of nanomaterials. During the last two decades, many nanomedicines got clinically approved and many more are under investigation in which theranostic acts as carrier systems (Peer et al., 2007; Davis et al., 2008). Specifically, in biomedical domain, this combination of nanomaterial-based theranostics with immunotherapy has opened avenues in disease management (Kievit and Zhang, 2011; Kaushik and Nair, 2018; Nam et al., 2019). In nanotheranostic preparation, nanoparticles (NPs) are often loaded with therapeutic drugs, proteins, photothermal agents, imaging moieties, or immunoactive molecules. To achieve multitasking activities, the particle should display unique characteristic properties and tenability. Different steps to functionalize and tag molecules with NPs have gained prominence introducing new advantages of nanoformulation (Liu et al., 2018; Overchuk and Zheng, 2018). Depending on the route of administration, the NPs with attached recognizable ligands are readily taken up by the targeted cells following receptor-ligand interaction (Song et al., 2017). The NPs capability to flow along with bloodstream or obstruction during circulation depends on enhanced permeation and retention (EPR) effects. However, in most cases complex biological barriers encountered by NP limits the bioavailability of active agents as they get distributed to even undesired sites. Opsonization, blood platelets, protein plasma, coagulation factors, phagocytic system and cellular internalization all contribute to the formation of protein corona (layer of proteins adsorbed) on NP surface while passing through vascular compartment (Longmire et al., 2008; Blanco et al., 2015). Advertently, creating a high hindrance to activity and selectivity of the targeting ligands attached to the surface of NP by further reducing therapeutic outcome (Dutta et al., 2007; Salvati et al., 2013; Obst et al., 2017; Mendes et al., 2018). All of this unsolicited phenomenon can be dealt with by finely tuning the NPs to envelop drug from the reactive surrounding. Even the physiological conditions in which the tumor cells grow and proliferate has also been an important aspect of research. The tumor microenvironment (TME) is quite complex and different among tumor kinds thus greater challenges are faced by theranostic NPs during cellular penetration to deliver the cargo (Roma-Rodrigues et al., 2019). With continuous advancements of clinical oncology from the perspective of high throughput research, many biomarkers have been identified that are exclusively expressed on the tumor cells (Mendes et al., 2018). Few are found in typical TME, thus can be distinctively targeted by the NPs upon reaching the tumor site and significantly improving the treatment efficacy (Hamid et al., 2011; Yuan et al., 2016; Roma-Rodrigues et al., 2019). Biomarkers presented by tumor cell and their persisting microenvironment enforce the requirement to schematize non-invasive techniques through utilizing their property of acting as a receptor to various ligands (Yuan et al., 2016; George et al., 2019). This has been the foundation of nano-based objectives to achieve maximum tumor eradication.

For facilitating stimulation or suppression of immune responses, the NPs are combined with various type of immune system derived or based materials/immunogenic, like antigens, adjuvants, DNA/RNA sequence, peptides, interleukins, cytokines, the stimulator of interferon genes (STING), TLR antagonists, and indoleamine 2,3-diooxygenase (IDO) inhibitors, growth factors, etc. These immunomodulators subjected to design immunomodulatory nanoformulations either use standalone or combinational approaches. (Le et al., 2018; Gorbet and Ranjan, 2020). Some materials have intrinsic immunomodulation properties exhibiting basic characteristics and thus lead as an efficient material in synthesizing immunomodulatory cancer theranostics (Song et al., 2017). To further enhance the efficacy of immunomodulatory agents, few co-delivery agents have also been investigated. They intend to accompany and strengthen the activity of low response generating immunomodulators (Le et al., 2019). Substantially in immunological treatments for dealing with receptor expression level, non-invasive imaging techniques of ultrasound, Computer Tomography (CT), Magnetic Resonance Imaging (MRI), Positron Emission Tomography (PET), optical imaging and, fluorescence using probes and visualization features have turned out to be potentially viable platforms (Ehlerding et al., 2016; Zavaleta et al., 2018; Kasten et al., 2019). Moreover, multifunctional theranostics can help in observing the actual route of precision nanomedicines which when administered to screened tumors collectively segregated themselves as per enhanced EPR effect (Jiang et al., 2017; Nam et al., 2019). To gain information about real-time response and disease progression, molecular imaging has accounted to facilitate the elimination of ineffective treatment regimes (Ehlerding et al., 2016). Diagnosis involving therapeutic guidance with imaging tool, aids in monitoring engineered NP loaded with cargo. Further, revealing the steps covered by carrier system both inside out of the targeted site like uptake of nanomedicine and intratumoral distribution helps in systematically developing a standard protocol (Chen H. et al., 2017). If incorporated externally, the diagnostic compound should remain stable and protected until the acting constituent gets unloaded at the targeted site (Hossen et al., 2019). Hence, controlled optimization of NPs circumscribes the future of immunomodulating nanomaterial supporting both anticancer drug and bioimaging tracer with the ability to withstand the body’s internal pressure and other interactions.

The integration of cancer with immunotherapy has introduced investment as a burden for meeting cost-effective maximum therapeutic response and lower toxicity. Looking at the proficiency required in attaining the desired efficacy of immunologically active small molecules, various alterations in nanotechnology have been opted to achieve enhanced biodistribution, localization, and kinetics (Goldberg, 2015). A comparatively simple solution to modify nanomaterial as expected, nanotechnology has offered reliable perspectives of working on theranostic. Dealing with immunotherapy and diagnostics concurrently to meet the challenging demands of cancer could commence acceptable clinical results. Successfully planned and designed treatment may also deter the imperfections in clinics that critically lack analytical response uprooting variations in immunomodulatory nanomedicine (Chen H. et al., 2017). Additionally, attaining uniform NPs characteristics throughout the nanomedicine formulation when considering personalized treatment could be advantageous. Before radiating to approaches used in engineering cancer theranostic, lets dive into understanding the properties of different nanomaterial associated carrier systems closely.

## Classification and Characteristics of Nanomaterials for Cancer Immunotheranostic

Multiple novel platforms are gaining interest in elucidating sophisticated solutions to treat cancer with emerging nanotechnology connecting immune response (Waldman et al., 2020). Nanotechnology interventions can also augment flexible biomaterials with designs related to additive manufacturing (Ghomi et al., 2020). However, with technical advancements, control over physicochemical parameters such as size, surface functionalization, shape and, charge paramount stimuli response as well as loading efficiency of nanomaterials. Variation in properties influences the underlying fate and mechanism of NPs at the target site (Ferrari, 2010; Fang and Zhang, 2016). Surface modifications leading to structural changes can be attained through different constructions of layers at the surface, shell, or in the core of NPs. Such alterations are executed by processing through metal ions, electrolytes, and surfactants (Shin et al., 2016). Tumor penetration and distribution enhances at NP size of 100 nm or less. Representing good antitumor activity, NPs at a much smaller dimension of 30 nm have been reported to enter even the poorly permeable tumor (Cabral et al., 2011). Another property of shape and charge states a reliable predictor for designing improved nanocarriers with reduced toxicity. When studied with different nanoscale geometries, under typical conditions the nanodiscs with hydrophilic anionic charge of high aspect ratio were found to be more internalized in epithelial and immune cells as compared to nanorods. Also, larger nanodisc and nanorods showed better uptake than smaller counterparts (Agarwal et al., 2013). The ratio of positive and negative surface charges for monodispersed and stable particles has also been evaluated as a deciding factor depending on the nature of the cargo to be delivered *in vivo* (Kranz et al., 2016). Functionalization of the material surface has been another important aspect to achieve effective targeting enabling binding of many ligands confronting optimum blood clearance and therefore sustainable half-life (Longmire et al., 2008; Fang and Zhang, 2016).

Like traditional material classification, immunomodulatory nanomaterials for cancer theranostics can also be broadly divided into three categories based on the core materials used: inorganic, organic (Mendes et al., 2018), and inorganic/organic hybrid NPs (Vivero-Escoto and Huang, 2011; Liang et al., 2013). Accompanied by considerable delivery criteria, drug delivery attempts to expedite synthesized multimodality nanosystems by controlled selection of NPs attributes. Despite such achievable goals, there is a need to optimize material choice depending on the type and nature of the drug delivery system for cancer immunotherapy.

### Inorganic Nanoparticles

Inorganic nanoparticles have exhibited an excellent potentials in the field of cancer theranostics with versatile functionalization properties. Gold, silver, iron-based NPs, quantum dots, etc. are widely studied as some important classes of inorganic NPs (Mendes et al., 2018). They have largely been explored for their imaging, magnetic, radiation-controlled, and hyperthermic properties (Mendes et al., 2018). Apart from being bioinert, gold demonstrates the ease of processing by surface modification and can be crafted into different shapes and sizes. Gold NPs with their intrinsic optical properties are being used in therapeutic and diagnostic applications (Huang et al., 2007; Shi et al., 2014). Due to this, gold NPs have mostly been investigated for both hyperthermia based therapeutic treatments and contrast based imaging using MRI and PET/CT scans (Medina-Reyes et al., 2017). Moreover, gold have turned out to be the most explored metallic NPs that can stimulate the immune system and deliver drugs, antigen, nucleotides, aptamers, etc. The second type of inorganic NPs, quantum dots (QDs) are conventional yet effective in its applciations. They represent nanosystems in the range of 2–10 nm built by different materials including carbon, zinc sulfide, titanium oxide, etc. They generally pack into layers giving the appearance of small dots that can be easily coated on other nanosystems forming good imaging agents (Medina-Reyes et al., 2017). With the currently available methods including green synthesis it has become possible to control size, functionalize, and modify QDs. Iron Oxide NPs hold an important position in the field of nanotechnology for generating radiofrequency and magnetic effect-based hyperthermia which is sublethal at temperature <43°C. Surface modification and high contrast properties of iron oxide NPs give easy access to imaging and diagnostics purposes with MRI and CT scans. Application has been observed in initiating immunotherapy simultaneous to activation of heat shock proteins enhancing immune responses (Medina-Reyes et al., 2017; Singh et al., 2019). Radionuclide in medicine is known as radio nanomedicine abd use radiolabeled nuclei constituting a special group of inorganic NPs. They are used as molecular imaging modalities in PET, X-Rays and Single Photon Emission Computed Tomography (SPECT) for bioimaging the target sites and photodynamic therapy (PDT). Radionuclides like Gadolinium, Hafnium, Yttrium, Lutetium have entered clinical trials as theranostic systems for radiotherapy providing treatment in combination with immunologic agents (Ferreira et al., 2019). Biocompatible silica NPs are another class of inorganic materials having size ranging from 1 to 200 nm. They have been explored in many nanosystems in the form of rod-like, non-spherical shaped, and mesoporous silica NPs. High loading capabilities, easy surface modification, and targeting methodologies add to their characteristics. Further, they are widely being used for bioadhesives, drug delivery, tissue imaging, and diagnosis purposes (Medina-Reyes et al., 2017; Xu et al., 2017). In cancer immunotherapy, porous silica and gold for immunomodulatory activity have been immensely studied. (Nguyen et al., 2012; Almeida et al., 2014; Shahbazi et al., 2015). Besides all the benefits of theranostic, inorganic NPs possess major challenges of toxicity and accumulation in the body, rendering the preference to organic over inorganic materials (Mendes et al., 2018). Facts have been supported by the reports suggesting superparamagnetic iron oxide and quantum dots for causing cancer as they induce immunogenic toxicity in few animal models (Kodali et al., 2013; Mashinchian et al., 2014).

### Organic Nanoparticles

With distinctively achievable morphology, excellent biocompatibility and lower toxicity organic NPs have been considered in drug delivery, imaging, and phototherapy for ages. Construction and tailoring of organic NPs can be done through hydrogen bonding, van der Waals, and electrostatic interactions (Fang et al., 2020). They form multiple types of material systems. Conventionally, lipid-based NPs are self-assembled vesicles, consisting of phospholipid amphiphilic molecules which serve as good carriers to deliver huge payloads. Improvement of lipid NPs can be easily done by controlling release rate, functionalizing with PEG for prolonged-release, targeting ligands, and fluorophores in response to some environmental stimulus (Hossen et al., 2019). They can be modified into different carriers like liposomes, micelles, niosomes, exosomes, etc (Medina-Reyes et al., 2017)**.** Highly biocompatible liposomes have been encapsulated with various drugs, imaging, photothermal, photodynamic agents, along with other immunological agents to increase the internalization, retention, and delivery properties of the nanoformulations (Mendes et al., 2018). Apart from lipids, various types of NPs include polymers assemblies. Nanospheres, nanocapsules, dendrimers, micelles, co-polymers conjugates, polymer-lipid conjugates, etc. are few polymeric delivery systems. Polymers have some significant properties like a smooth escape from retinoendothelial system, high loading capacity, stimuli-based release of cargo from the depot and safety. Flexibility in design, diversity and large-scale synthesis makes polymers stand ahead of other organic NPs (Twibanire and Grindley, 2014). Polyethylene glycol (PEG), Polyethylene-L-glycolic acid (PLGA), and Polylactic acid (PLA) are some Food Drug Administration (FDA) approved polymers widely being used in nanoformulations. Nanosystems explicitly imposing properties like good mechanical strength use polymer mixtures to attain desired synergistic physiochemistry and sustainable dependency. A variety of copolymers, metals, and nucleotide-based conjugates are being introduced into multiple approaches (Mendes et al., 2018). Recently, multinuclear polymer has been developed for increasing protein loading capacity and release in a sustained manner without compromising bioactivity (Wu et al., 2014). As another type of organic NPs, carbon-based materials have been classified based on structures, like nanotubes, graphene sheets/quantum dots, fullerenes, etc. They depict unique structural rigidity and electrical properties (Georgakilas et al., 2015). However, studies have shown potential toxicity even at lower carbon concentrations restricting nanotheranostic prerequisites where it fails safety concerns (Medina-Reyes et al., 2017). The naive yet important group represents biomolecule based materials that use biological building blocks like carbohydrates, DNA and peptide as NPs aiming to induce minimum systemic toxicity with optimum desired immune response. Recently evolved and extensively exploring biological derived cell membranes or camouflages have been harnessed as vehicle system. Due to natural extraction, such bounded systems survive for longer in the biological fluids without losing stability and has been reported to easily navigate inside the targeted site (Mendes et al., 2018). However, during the material selection of organic nature, most commonly used and widely preferred are biodegradable lipids and polymer-based NPs (Shargh et al., 2016).

### Inorganic/Organic Hybrid Nanoparticles

Overcoming the major drawback of toxic inorganic biomaterial by cheap and simple green chemistry approach has increased their utilization in hybrid systems along with organic materials (Madamsetty et al., 2019). Sufficing the advantages and disadvantages of each other, incorporation of inorganic and organic NPs in single system has added to the cancer immunotherapy development schemes. They use many stimuli-responsive nanocarriers demonstrating controlled drug release profile assisted by external triggers like ultrasound, heat, temperature, pH, magnetic field, and electric field (Mi, 2020). For instance, apoferritin, a protein nanocage having iron at its core shows pH-dependent structural assembly. This fundamental behavior is being highly appreciated in cancer theranostics (Madamsetty et al., 2019). This mixed type NPs can also be used to address covalent and non-covalent linkages with the drug. Superparamagnetic iron oxide NPs in association with polymer or lipid vesicle carriers can gauge the release of drugs under external magnetic field (Alonso et al., 2016; Grillo et al., 2016; Li et al., 2019). Using the ablation property of inorganic NPs like Gold and advantages of Iron oxide with biocompatible characteristics of polymer, a combinational systems have been synthesized and studied for near infrared (NIR)-triggered chemo-photothermal therapy (PTT) (Chen C. W. et al., 2017).

Porphyrin NPs of perfluorocarbon gas-filled lipid shells can be stimulated by low-frequency ultrasound which also acts as photoacoustic and fluorescent contrast imaging agent (Huynh et al., 2015). Magnetic nanogrenades with pH-sensitive ligands have been developed from iron oxide self-assembled NPs which disassemble into highly active subcellular compartments enabling good MR contrast, photodynamic/thermal, and fluorescence activity to detect early stage cancer. This has ventured as effective treatment for drug-resistant neoplastic cells (Ling et al., 2014). A liposomal carrier with ZnS/ZAISe QDs embedded in a phospholipid bilayer, loaded with DOX and later fused with macrophage membranes has shown superior optical imaging while accumulating in lung lesion tissues of mice (Liang et al., 2020). The modern hybrid systems would integrate physicochemical and bio-functionalities for supporting co-therapies by conjugating varieties of therapeutic cargos (Fan et al., 2017). Altogether, looking ahead of these entire advents, hybrid-based smart nanomaterial system could produce safer and superior clinical results with remotely supervised therapeutic, diagnostic and monitoring (Thorat et al., 2019).

To summarise, NPs demonstrate the ability to penetrate inside the tissue via the leaky vasculature and increase the EPR effect. Smaller molecules tend to accumulate faster in tumors and can stay for a longer time. Functionalized NPs could achieve active targeting in the tumor site with the help of proteins, aptamers, peptides, carbohydrates, nucleic acids, etc (Shargh et al., 2016). A sustained and controlled release can be attained effortlessly by using polymeric NPs because the drug release is either via diffusion, swelling, or via bulk erosion in a time-dependent manner. Properly engineered biodegradable nanomaterials have benefits such as maintaining the drug concentration in the desired range for long periods (Singh et al., 2019). The most prominent property of NPs is the capability to resemble multifunctionality and could be beneficial for immunological cancer research. The intention has been to incorporate imaging agent for monitoring the effect of treatment so that précised amount of drug is delivered at the correct location. Table 1 enlists examples of theranostic nanomaterial for delivering or augmenting immunomodulatory effects studied on cancer models.

**TABLE 1:** List of Theranostic nanomaterials for immunomodulation activity.

Types of nanomaterials/delivery platforms		Immunoactive agent	Size	MODEL/TARGET	References
Inorganic nanoparticles	Nanosized metal-organic frameworks (nMOFs) loaded with doxorubicin	FITC-labeled antiHER2/neu	∼120 nm	BT-474 and MCF-7 cells	Cherkasov et al., (2019)
	Iron oxide nanoparticles	Hyaluronic acid (HA)	∼80.9 nm	CD44-overexpressing cancer cells/MDA-MB-231 cancer cells	Soleymani et al., (2020)
	RGD-modified dendrimer-stabilized gold nanostars (RGD-Au DSNSs)	Small interfering RNA (siRNA)	55.1 ± 12.6 nm	*Cancer* cells overexpressing α_v_β_3_ integrin	Wei et al., (2016)
	Spiky gold nanoparticles (SGNPs) coated with PDA sub-therapeutic dose of doxorubicin	-	150 ± 45 nm	CT26 colon carcinoma, TC-1 submucosa-lung metastasis, advanced head and neck squamous cell carcinoma (HNSCC)	Nam et al., (2018)
	Gold nanoparticles (AuNPs)	Hyaluronic acid (HA) and antigen (ovalbumin, OVA)	∼175 nm	CD44-positive B16 melanoma cell	Cao et al., (2018)
	Thiolated-polyethylene glycol (PEG) coated AuNPs	TAMs-targeting peptide (M2pep) and thiolated anti-VEGF siRNA labeled with alexa Fluor^®^ 488	≈15 nm	A549- lung adenocarcinoma tumor	Conde et al., (2015)
	Chitosan-coated hollow copper sulfide (CuS) nanoparticles	Oligodeoxynucleotides cytosine guanine (CpG) motifs	∼85 nm nm	EMT6 tumors	Guo et al., (2014)
	PEGylated ferrimagnetic vortex-domain iron oxide nanoring (FVIO)	anti-PD-L1	∼150.9 nm	4T1 breast tumor	Liu X. et al. (2019)
	PEGylated pure FeNPs	Anticytotoxic T lymphocyte antigen-4 (anti-CTLA4)	30–50 nm	4T1 breast tumor	Chao et al., (2019)
	3-Aminopropyltriethoxysilane (APTES)-modified Fe_3_O_4_ nanoparticles (FeNPs)	Cytosine-phosphate-guanine (CpG)	34.5 ± 5.0 nm	C26 colon cancer and 4T1 breast tumor	Zhang Xue. et al. (2018)
	ETP-PtFeNP loaded-oxaliplatin (IV) prodrug	α-enolase targeting peptide	∼ 24 nm	4T1 breast tumor	Chen et al., (2019)
Organic nanoparticles	CPCI-NP loaded with doxorubicin (DOX)	anti-PD-1 monoclonal plus anti-CTLA4	∼20 nm	Orthotopic OSC-3 oral cancer xenograft model	Zhang L. et al. (2018)
	Glucocorticoid-induced TNF receptor family related protein/poly (lactic-co-glycolic acid) (GITR-PLGA) nanoparticles loaded with IR-780 dye	Imatinib	^a^	B16/BL6 and MC-38 tumor-bearing mice	Ou et al., (2018)
	Benzoporphyrin derivative (BPD) poly (lactic-co-glycolic acid) nanoparticles	Cetuximab photo-immunoconjugates onto FKR560 dye	∼100 nm	Ovarian cancer and glioblastoma	Huang H. C. et al. (2018)
	Monoclonal antibody (mAb)-photosensitizer conjugated with liposomal daunorubicin	Panitumumab (*Pan*) conjugated to IR700 fluorescence dye	^a^	EGFR-negative Balb3T3/DsRed cells	Sano et al., (2014)
	a-MBM-DOX nanosystem	anti-MDR1 molecular beacon (MB)-based micelle (a-MBM)	∼20 nm	OVCAR8/ADR cells	Zhang R. et al. (2017)
	Acetylated fucoidan (AcFu) nanoparticles loaded with DOX	Fucoidan	∼137.5 ± 2.475 nm	HCT-116 and HCT-8 colon carcinoma cells	Lee et al., (2013)
	Hematoporphyrin monomethyl ether (HMME)/R837- sonosensitizer co-encapsulated liposomes	anti-PD-L1 and imiquimod (R837)	∼157.3 nm	4T1 breast tumor	Yue et al., (2019)
	PEGylated-CyI nanocarriers	Hyaluronic acid	112.5 ± 1.8 nm	4T1 breast tumor	Chi et al., (2020)
	DNA nanotrain (aptNTrs)	sgc8 aptamer	^a^	T-cell acute lymphocytic leukemia (CEM) cells	Zhu et al., (2013)
	High-density lipoprotein mimicking magnetic nanostructures (HDL-MNSs)	HDL	41 ± 19 nm	SR-B1 positive B-cell lymphoma	Singh et al., (2019)
	POP micelles	siRNA	87.5±1.5 nm	B16-F10 melanoma	Wang et al., (2016)
	Poly-lactic-glycolic-acid (PLGA) NPs conjugated with polyDopamine	Hepatitis B surface antigen and TLR9 agonist unmethylated cytosine-guanine (CpG) motif	910.52 ± 5.64 nm	Bone marrow-derived dendritic cells	Liu et al., (2016)
Hybrid nanoparticles	Tantalum oxide (TaO_x_)-Cy7-DOX-PEG NPs	HA	∼37.5 nm	MDA-MB-231 breast tumor	Jin et al., (2015)
	polyfunctional gold-iron oxide nanoparticles (polyGIONs) coated with β-cyclodextrin-chitosan (CD-CS) hybrid polymer co-loaded temozolomide (TMZ)	miRNA (miR-100, antimiR-21) and PEG-T7 peptide	∼53.1 nm	Glioblastoma	Sukumar et al., (2019)
	Calcium bisphosphonate (CaBP-PEG) nanoparticles radiolabeled separately with99mTc and32P	Bisphosphonates	∼50 nm	4T1 breast tumor	Tian et al., (2018)
	Multifunctional doxorubicin-loaded fucoidan capped gold nanoparticles (DOX-Fu AuNPs)	Fucoidan	∼83 nm	MDA-MB-231 breast tumor	Manivasagan et al., (2016)
	Fe_3_O_4_ magnetic nanocluster (NC) in leukocyte membrane	PD-L1 antibody; TGF-β inhibitor	^a^	B16F10 melanoma tumor	Zhang et al., (2019)
	Gold nanorods loaded with doxorubicin	TLR9-specific unmethylated oligodeoxynucleotide CpG	∼91.5 nm	H22 hepatocellular carcinoma	Tao et al. (2014a)
	Lipid-protamine-DNA nanoparticle	anti-PD-L1 mAb	∼129 nm	CT26-FL3 colorectal tumor	Song et al., (2018)
	Polyethylene glycol (PEG) and polyethylenimine (PEI) dual-polymer-functionalized graphene oxide (GO-PEG-PEI)	TLR9-specific unmethylated oligodeoxynucleotide CpG	^a^	CT26 colon tumor	Tao et al. (2014b)
	Chlorin-nMOF	Heavy metal cluster secondary building units (SBUs) and IDO inhibitor (IDOi)	∼83.2 and ∼72.7 nm	CT26 and MC38 colorectal tumor	Lu K. et al. (2016)
	FeCO-DOX-MCN mesoporous carbon nanoparticles	Triiron dodecacarbonyl (FeCO)	198 ± 10 nm	MCF-7 breast tumor	Yao et al., (2019)
	Iron oxide (IO) and paclitaxel (PTX) encapsulated PLGA–PEG nanomicelles	Self-peptide	106 ± 1.23 nm	RAW264.7 cells and S180 sarcoma tumor	Zhang K. L. et al. (2017)

^a^Data not available/distinctly reported.

## Approaches for Cancer Immunomodulated Biomaterial System

**FIGURE 2:**
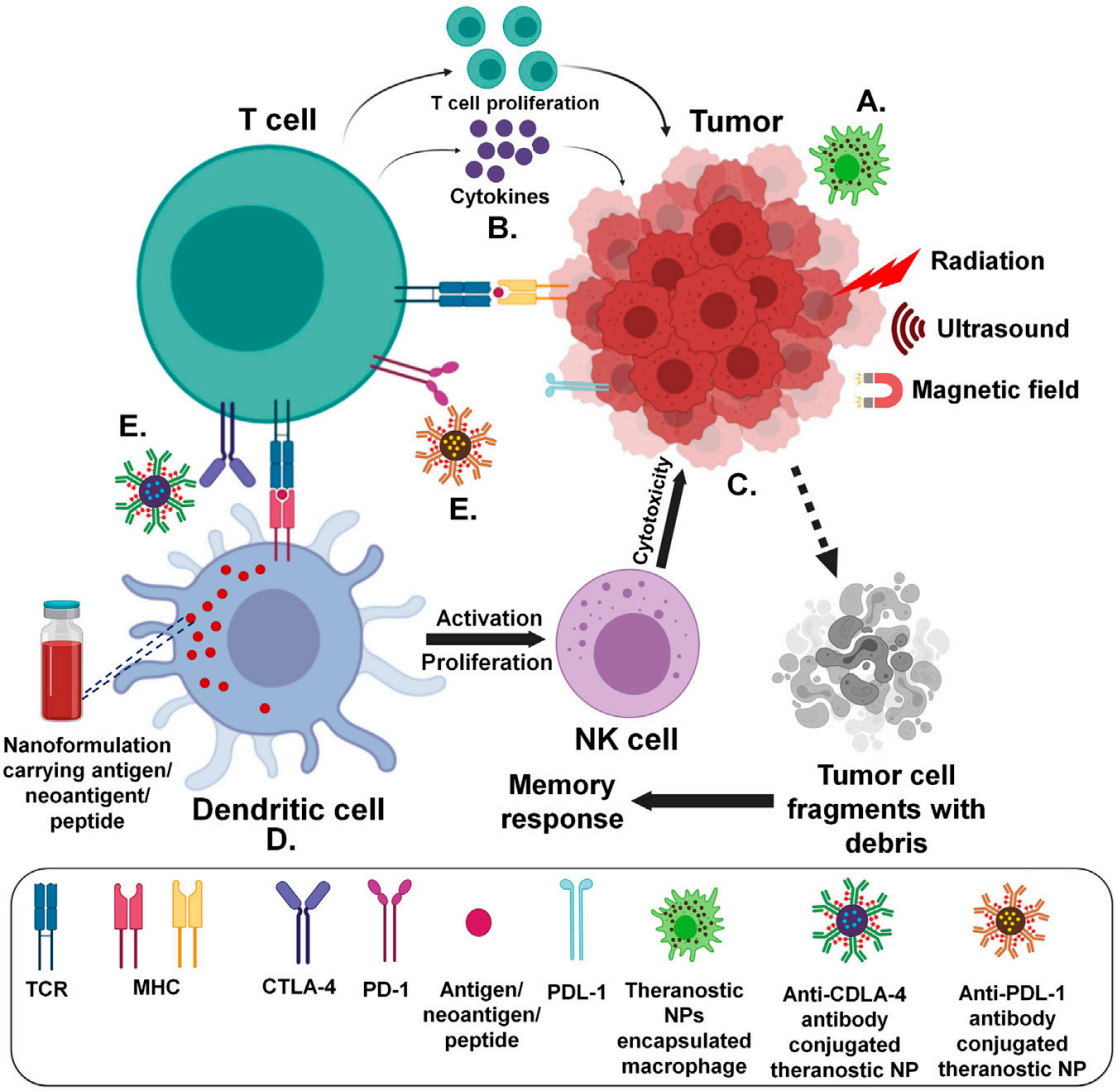
Strategies for antitumor immune system targeted delivery of theranostic NPs (Created with BioRender.com) **(A)** Immune cell/membrane entrapped **(B)** Direct/indirect cytokine release assisted **(C)** Tumor microenvironment and components targeted **(D)** Utilizing antigen presenting cells **(E)** Antibody mediated.

Malignant cells in the body have ability to proliferate uncontrollably and eventually evade detection by immune system as they genetically modify themselves and continuously recruit immune cells for their growth and development (Gonzalez et al., 2018). However, certain strategies can be adopted to enhance the potential of immune system in fighting back robustly with well-controlled therapeutic approaches based on advanced delivery systems as summarized in Figure 2 (Ridge et al., 1998; Jain et al., 2015; Chen Q. et al., 2016; Deng and Zhang, 2018; Kutova et al., 2019; Chulpanova et al., 2020; Hauge and Rofstad, 2020). Specifically, functionalized biomaterials by physical or chemical bio-conjugation can help to suffice the need of treatment requirements. Enhancing or suppressing the immune response through biomedical involvement can diversify choices in regenerative medicine and cancer immunotherapies (Kim et al., 2020). Selections based on the type of immunomodulation assisted by biomaterial carrier such as a NP with desired properties can be achieved by various methods.

### Immune Cell Utilization

**FIGURE 3:**
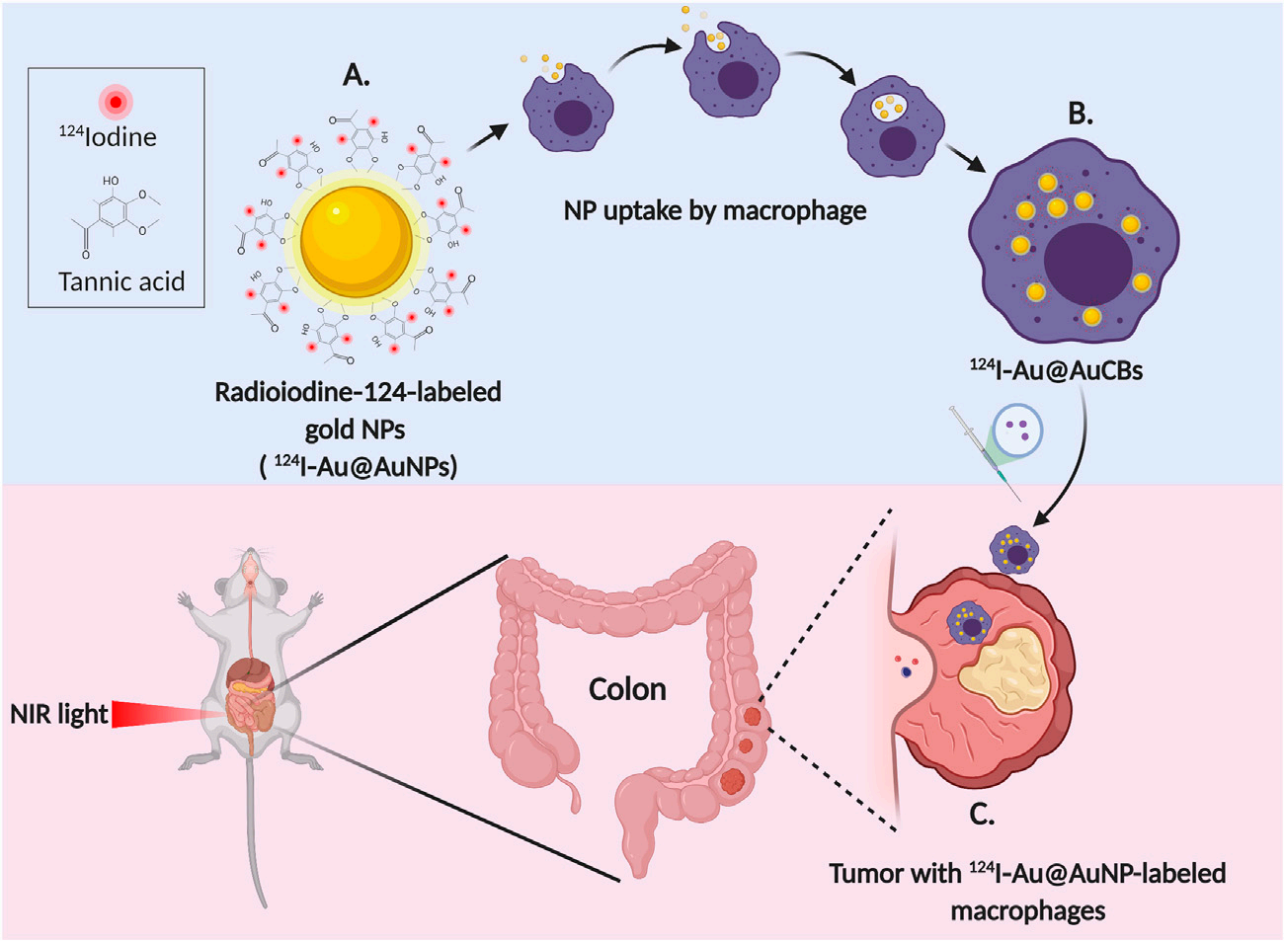
Macrophage mediated photothermal therapy and imaging of colon cancer **(A)** Radioiodine-124-labeled gold nanoparticles with crushed gold shells (124I-Au@AuNPs) **(B)** Uptake of 124I-Au@AuCBs by macrophage **(C)** Ablation and imaging of the tumor (Created with BioRender.com).

Direct entrapment inside the cell, cell membrane-derived biomaterial coating or delivery by surface attachment provides new and interesting areas of investigation in dealing with cancers effectively (Tan et al., 2015; Fang et al., 2018; Li R. et al., 2018; Li Y. et al., 2019). Immune cells have been incorporated widely as delivery agents for treating many cancers (Wayne et al., 2019). Inhibiting the progression of cancer through the neutralization of circulating tumor cells has also been studied. Apart from engineering nanomaterials for conjugation and directional chemical modifications; advantages of cellular components involved in defense mechanism of the body have been investigated. Kang et al., used drug-loaded poly (lactic-co-glycolic acid) NPs enveloped by neutrophil membranes for managing metastasis by preventing niche genesis (Kang et al., 2017). In another study conducted by Coa et al., a targeted delivery system with NPs of poly (ethylene glycol) methyl ether-block-poly (lactic-co-glycolic acid); PEG-PLGA conjugated celastrol drug further coated by neutrophil membranes were employed to treat pancreatic carcinoma. Neutrophil membrane coated NPs reported to assist in site-specific distribution improving bioavailability. The NPs accumulated as first-line recruitment in the site of inflammation assisting cytokine release (Cao et al., 2019). Recently, one more study was conducted to inhibit cancer by the process of natural destruction mechanism. A nanosized delivery system with Cisplatin loaded pathogen secreted vesicles was recognized and engulfed by neutrophils. The chemical drift generated by the inflamed tumor caused neutrophil migration and subsequent infiltration at the tumor site releasing NPs. Enhanced targeting efficiency and its combination with PTT was shown to effectively irradiate tumor in EMT6 tumor-bearing mice (Li et al., 2020). Macrophages have a better storage capacity and can easily penetrate the solid mass as the tumor progresses rendering them as better drug delivery vehicles. They can even serve to activate pro-drug into active moiety (drug) at the site of release. Miller et al. showed the activated release of fluorescent Platinum (IV) prodrug conjugated with poly (D,L-lactic-*co*-glycolic acid)-*b*-poly (ethylene glycol); PLGA-b-PEG by tumor associated macrophages (TAM). The PLGA-b-PEG self-assembly consisted of a hydrophilic PEG shell to enhance the circulation time of unreacted Pt compound and PLGA formed the hydrophobic core of NPs. Imaging was assisted by a fluorophore which acted as an *in vivo* image analyzer enabling visualization of controlled drug release kinetics by TAM (Miller et al., 2015). Another experiment using fluorescent imaging was performed by Sehwan et al. and co-workers to show the effective uptake and enhanced bioavailability of Ag NPs by macrophage deployed as delivery vehicle (Kim et al., 2020). Polymeric NPs carrying IL-12 cytokines demonstrated the modification from M2 to pro-inflammatory M1 type macrophages (Wang et al., 2017). Engineered nanomaterials have become the latest emerging area of research for inducing antitumor biological response by stimulating immune cells. In a recent experiment, NP assisted artificially reprogrammed machinery was used to not only enhance the macrophage intrinsic activity for effective tumor targeting but were also proposed to develop intramural immunosuppressive resistant macrophages (Li et al., 2019). Hyaluronic acid (HA) with its high affinity toward CD44 protein rich in macrophage was coated on superparamagnetic iron oxide NPs. Macrophages with hyaluronic acid-superparamagnetic iron oxide nanoparticles (HIONs) showed antitumor action by chemotaxis and magnetic movement. HA acted as a medium for effective internalization and chemical signaling response whereas, iron oxide as a mode of activation through magnetic trigger. Both of these examples demonstrated the production of anti-inflammatory factors for tumor repression by rehabilitating M2 to M1 phenotype (Fang and Zhang, 2009; Li et al., 2019). In a similar study, macrophage-mediated PTT assisted by radioiodine-124-labeled gold NPs with crushed gold shells (124I-Au@AuCBs) was examined for colon cancer in mice. It was noted that 124I-Au@AuCBs were retained when taken up by macrophages and hence imaging of the tissues was possible. Ablation of the cancerous tissues substantiated results that the therapy had a significant antitumor effect. The scheme is shown in Figure 3 (Lee et al., 2019).

T cell plays an important role in immune response generation and cancer immunomodulation. One way to achieve T cell activation is by the active cross-presentation of antigen (Tumeh et al., 2014; Rosenberg and Restifo, 2015). For effective cytosolic delivery of antigen to antigen-presenting cell (APC) and then to the targeted cells various NP delivery systems are being explored. PC7A copolymeric NPs labeled with indocyanine blue as an imaging agent have been reported to be an effective tumor antigen delivering agent attaining massive T cell response. This was the cause of increased surface presentation and activation of type I interferon-stimulated genes. Also, when combined with anti-PD-1 antibody, PC7A nanovesicles have shown a significantly higher survival rate in TC-1 tumor model (Luo et al., 2017). The migration, distribution, and kinetics of T-cells can be visualized by labeling with gold NPs as a contrast agent and green fluorescent protein for fluorescence imaging to better understand the physiological interactions between T-cells and tumor cells (Meir et al., 2015). In a study, amphiphilic micelles of conjugated curcumin-polyethylene glycol (CUR-PEG) natural polyphenols were delivered to the tumor site in a murine melanoma model. Combination of CUR-PEG increased CD8^+^ T-cell cytotoxicity, tumor necrotic factor -α and IFN-γ, with declination of regulatory T-cells (Tregs) was observed (Lu Y. et al., 2016). NK cells are being explored widely in cancer immunotherapy due to their unique feature of distinguishing normal and tumor or infected cells. Unlike T-cell, NK cells don’t rely on tumor antigen or activation response. Ease of extraction, broad specificity, absence of side effect and quick response influence NK cells to adopt with other anticancer therapies (Sun et al., 2009; Vivier et al., 2012). Certain transforming growth factors (TGF) released from tumors inhibits NK cell production and responses. A therapeutic approach has been investigated to downregulate the immunosuppressive signaling pathway blocking the activity of TGF. A Manganese oxide-based nanoparticle system synthesized by reduction and complexation with siRNA has been recently reported. NPs binding to TGFBR2 receptor lead to TGF inactivation causing non-interference in NK cell expression (Adjei et al., 2019). In another research, epidermal growth factor receptor (EGFR) bearing tumor targeted multifunctional engineered PEG-PLGA biocompatible NPs encapsulating drug and NK cell activating agents were nanoconjugated. These NPs designed for controlled release upon systemic administration resulted in activation and accumulation of NK cells mediating antitumor responsive chemoimmunotherapy (Au et al., 2020).

### Internalization of Immunostimulatory Cytokines

Long since the role of cytokines manifested in cancer there have been many effective developments as the anticancer advent commenced (West, 1989). The involvement of cytokines in immune cell activation and differentiation has improved the chances of exploration to molecular basis (Lee and Margolin, 2011; Chulpanova et al., 2020). Cytokines including interferons (IFN-γ and IFN-α) and interleukins (GM-CSF, IL-7, IL-12, IL-15, IL-18 and IL-21) can act as competent antitumor mediators whereas, pro-inflammatory cytokines like IL-1, IL-6, IL-10, TNF-α, and TGF-β suppress the immune response thus retarding the antitumor activity (Lee and Margolin, 2011; Burkholder et al., 2014; Yan et al., 2015; Conlon et al., 2019; Chulpanova et al., 2020). IL2 has been investigated for the activation and expansion of NK cells, T-cells, NKT-immune cells (Chung et al., 2014; Boyiadzis et al., 2017; Exley et al., 2017; Yoshida et al., 2017). Individual target cancer immunotherapies involving either stimulation of cytokines antitumor effect or negative regulation of pro-inflammatory immune-suppressive action will assist in enhancing analysis. However, the loopholes of unavoidable systemic pro-inflammatory effects limit cytokines as delivery agents requiring a much more versatile delivery system (Michallet et al., 2004; Pachella et al., 2015). It is also important to include these treatment strategies in combination with immunotherapy to limit toxicity (Melero et al., 2015). In one experiment, cationic polyethylenimine PEI and hydrophilic polyethylene glycol coated dendritic mesoporous silica nanoparticles (PEI-PEG-DMSN) were used to encapsulate TNF-α which had earlier reported to cause undesirable cytotoxicity on normal cells when administered systemically. The pH- responsive copolymer of PEI-PEG carrier system helped in releasing the cytokine cargo into the tumor cell due to acidification digestion. DMSN conjugation with different dye labels of Rhodamine B, Fluorescein and Pacific Blue, tracked delivery and cellular uptake pathway of TNF-α inside the cancerous cell (Kienzle et al., 2017). In another study, hydrophobic TGF- β receptor 1 antagonist was solubilized in methacrylate conjugated β cyclodextrins (CD) which contained hydrolysable ester bond between succinylated-CD and methacrylate group. This in conjunction with a liposome-encapsulated biodegradable polymeric matrix of PLA-PLG through covalent bond facilitated the sustained release of entrapped IL-2 and CD loaded TGF- β. Imaging was carried by rhodamine complexed with functionalized β cyclodextrins (Park et al., 2012). CD137, a member of TNF activates co-stimulatory signal generation of T-cell (Mittler et al., 2004). Liposomes are highly efficient carriers of cytokines (Siegler et al., 2016). Studies have shown that encapsulating IL-2 in multilamellar liposomal carriers significantly increases the circulation times along with decreased hematologic toxicities. The liposomes were able to retain the biologic activity of IL-2 and ensured the same effect while administering 7.5 times higher the dose of free cytokine indicating increase in tissue internalization (Anderson et al., 1992; Siegler et al., 2016). A study to overcome the systemic immunotoxicity of cytokines and circulating lymphocytes was performed through localized delivery involving thiol linkage of IL-2 and anti-CD137 antibody Fc region on the surface of PEGylated Liposome. Here, esterification between InfraRed fluorescent dye and IL-2 or anti-CD137 resulted in tracking tumor penetration (Zhang Y. et al., 2018).

IFNs have well been known in controlling cancers like Kaposi’s sarcoma, melanoma, kidney cancer, and hairy leukemia due to their ability to dually unleash an anti-tumor response and control tumor cell proliferation (Maeda et al., 2014). NPs having an affinity for the anionic membrane of mitochondria, based on a biodegradable poly (lactide-co-glycolide)-b-polyethyleneglycol (PLGA-bPEG) copolymers as a carrier system bonded with triphenylphosphonium (TPP) cation were prepared and linked with zinc phthalocyanine photosensitizer. The system after cascading mitochondria induced apoptotic cell death due to targeted light stimulated delivery of cargo enabled secretion of IL-18 along with IL-12, which eventually led to the production of IFN-γ by DC (Marrache et al., 2013). In a research, silica modified gold nanorods incorporated in cytokine-induced killer (CIK) cells were used for gastric cancer. Through *in vivo* experiments, it was observed that 4 h post-injection, these CIK cells labeled with gold nanorods could actively target gastric cancer cells MCG803 and image simultaneously using photoacoustic based imaging. An upregulation of cytokines such as IL-1, IL-12, IL-2, IL-4, IL-17, and IFN-γ was observed along with the eradication of gastric cancer tissues by PTT under NIR laser radiation (Yang et al., 2016). Another group studied the combinatorial effect of immunologically active agent cytokine IL-12 and monoclonal antibody cetuximab to treat head and neck carcinoma thus limiting the individual toxicity of cetuximab and IL-12. Preclinical hypothesis suggested safe antitumor action with enhanced immune response by secretion of IFN-γ apart from inducing NK cell-mediated antibody-dependent cellular cytotoxicity (ADCC) (Mcmichael et al., 2019). Comprehensively, cytokines can be delivered efficiently with the help of NPs since they get degraded and cleared rapidly from the system. It is also due to NPs passive accumulation in the leaky vasculature of tumor (Siegler et al., 2016).

### Targeting the Tumor Microenvironment

The tumor microenvironment (TME) acts as a major barrier in reaching the targeted site whose negligence leads to inefficient cellular uptake of NPs and connected active moieties accompanying therapy failure. Cancerous heterogeneous cellular complexation with fibroblasts, adipocytes, myofibroblasts, the extracellular matrix (ECM) along with immune cells and the vasculature system complicate the treatment (Albini and Sporn, 2007; Chen et al., 2015). Also, the continuous addition of cells through the upregulation of proangiogenic proteins such as vascular endothelial growth factor (VEGF) brings about endothelial cell migration and proliferation causing an excessive accumulation of endothelial and abnormal perivascular cells in the area of the tumor. Abnormal vascularisation prohibits adequate penetration of anti-cancer agents into the desired site of action ending up in minimal anti-cancer effect (Banerjee et al., 2011; Cesca et al., 2013; Khawar et al., 2014; Klemm and Joyce, 2014). To circumvent this obstacle, various nontoxic biocompatible nanoparticulate delivery systems have been developed. One such stratagem employed by Sengupta et al. involved the development of poly-(lactic-co-glycolic) acid (PLGA) polymeric nanoparticles, where Doxorubicin was covalently bound to the inner core of NP followed by the encapsulation of combretastatin within the outer lipid envelope. On delivery with PLGA (nanocell), combretastatin being an anti-angiogenic agent disrupted cytoskeletal structures leading to a dysfunctional vascular system in TME. This disorientation assisted the unhindered easy entrapment of Doxorubicin inside the cancer cell prolonging therapeutic release for efficient apoptosis by DNA intercalation as compared to only the liposome or the free drug (Sengupta et al., 2005). Anti-fibrotic and extracellular pH targeting can also potentiate cancer therapies like chemotherapy or radiation targeting TME thus stalling tumor progression (Li X. et al., 2017; Hauge and Rofstad, 2020). Solid tumors involve a stiffer ECM which doesn’t allow sufficient penetration of drugs due to the presence of many components. Alteration in one such major constituent, collagen affects tumor cell migration, nutrition supply and oxygen accessibility. Lipoxygenases (LOX) enzymes or matrix metalloproteinases such as LOX-like protein (LOXL_2_, LOXL_4_, MMP_2_, MMP_9_ and MMP_14_) and growth factors inducing collagen deposition (eg, VEGF) are HIF-regulated genes and components that can help in remodeling the ECM and tumor fibrosis (Page-McCaw et al., 2007; Kondratiev et al., 2008; Liu T. et al., 2019). Hence, understanding the underlying modification mechanism of ECM components requires insight into sensitizing biomaterial delivery systems (Harisi and Jeney, 2015; Henke et al., 2020). Amphiphilic poly (D, L-lactide-co-glycolide)-block-poly (ethylene glycol); PLGA-b-PEG-COOH copolymer-based NPs were developed by solvent displacement method and then self-assembled for surface modification through carbodiimide chemical bonding covalently linked with LOX_AB_ (LOX inhibiting antibody). NP size of around 220 nm favored passive targeting of tumors, also the LOX_AB_ coating ensured retention and active targeting of the ECM. A higher therapeutic index of almost 50 doses was achieved with lesser processing steps as compared to the soluble anti-LOX anti-bodies without the nanomaterial coating (Kanapathipillai et al., 2012). Another component contributing to the 3D tumor environment in ECM is a polysaccharide, hyaluronic acid (HA). Apart from being utilized as an HA-delivery based platform various anti-HA approaches have been developed to increase tumor infiltration and easy penetration into the TME (Amorim et al., 2020). One such anti-tumor immunotherapy includes hyaluronidase (HAase) supplemented with DCs maturation potentiating PEI/CpG/OVA nanovaccine containing polycationic polyethyleneimine (PEI) delivery vehicle. The nanosystem carried ovalbumin and unmethylated cytosine-phosphate-guanine antigens simultaneously. Thus, provides a promising treatment regime favoring the importance of ECM disrupting agents (Guan et al., 2018).

Tregs are T-cells that prevent the onset of autoimmune diseases by generating tolerance toward autoantigens developing pro-tumorigenic TME. They have found to be associated with the upregulation of immunological biomarkers including immune checkpoint molecules, cytotoxic T-lymphocyte associated protein 4 (CTLA-4), glucocorticoid-induced TNFR family related gene (GITR) and certain T cell activation markers, CD25 and CD69 (Kakita et al., 2012; Schuler et al., 2012; Jie et al., 2013; Lin et al., 2013; Pedroza-Gonzalez et al., 2013; Sugiyama et al., 2013; Han et al., 2014; Scurr et al., 2014; Kurose et al., 2015). A PEG-modified single-walled carbon nanotube possessing glucocorticoid-induced TNFR related receptor (GITR) with labeled NIR-emitting fluorophore was able to efficiently target Tregs in a B16 melanoma model as compared to the splenic Treg or the intratumor non-Treg. Preferential increase in selectivity and efficiency due to the EPR effect along with use of biomarkers enriched in intratumor Treg recognition could be postulated for easy access to targeting T cells in the TME (Sacchetti et al., 2013). Another cell type in TME is Myeloid-derived suppressor cells (MDSCs) which do not mature into immune cells such as granulocytes, DCs, etc but are related to angiogenesis and metastasis (Shvedova et al., 2013; Park et al., 2018; Wang and Mooney, 2018). Indeed, these cells when present in the TME may suppress T cell proliferation and curb the activation of NK cells, while allowing Treg cells to differentiate. So, it becomes necessary to modulate T-cells and precisely design nontoxic NPs performing targeted cargo delivery which can be highly advantageous (Park et al., 2018). Gemcitabine, a potent anticancer agent when modified by Lauroyl contained in PEGylated lipid nanocapsules and tagged by a fluorescent dye, accumulated at the tumor site and spleen resulting in wipe-off populations of MDSCs in EG07- OVA tumors. Moreover, T cell proliferation and CD8^+^ T cell activation were reported, when the nanosystem was delivered prior to adoptive cellular therapy (Sasso et al., 2016).

### Delivery of Antigens for Immune Activation

Vaccines that can work effectively against cancer by stimulating or enhancing an immune response have been trending widely. Cancer vaccines are basically of two types, one is the preventive type and the other is the treatment-based vaccine. The vaccines administered usually are antigen/adjuvant vaccines, viral vectors and DNA vaccines, DC vaccines and whole-cell vaccines (Barra et al., 2019). Among them, DCs act by triggering immune mechanisms against tumors and have been investigated for their potential role in immunogenic vaccines. As primary antigen-presenting cells their major role is in generating an adaptive immune response. Various ways to enhance antigen uptake by DCs thus assisting better antigen recognition for T cell and natural killer cell activation have been explored (Ridge et al., 1998; Kalergis et al., 2001). Various events support evidence that neoantigens could be targeted for an effective antitumor immune response generation (Hu et al., 2018). To maximize the initial response of an anti-cancer immune system, the antigens must be delivered efficiently to lymph nodes for which NPs are being studied as vehicle (Tran et al., 2018; Zhang L.X. et al., 2018).The nanocarrier systems for antigen delivery must display some integral properties. Firstly, a medium-sized nanocarrier ranging from 5-100 nm could help in effective circulation and delivery through the lymphatic vessels to the lymph nodes. Secondly, particle shape turns to play another key role even in antigen delivery. Non-spherical NPs have higher aspect ratios, higher circulation times, better penetration abilities into tumors, and solid tissues along with prolonged margination effects (Manolova et al., 2008). Third, uptake of NPs by cells and activation of immune responses is surface charge-dependent. Positively charged NPs could cause an upsurge in immune responses as compared to negatively charged or neutral carriers. The downside to using them, however, is that they could get immobilized in the negatively charged ECM, reducing their tissue penetration capacities. On the other hand, hemolysis and platelet aggregation could occur in the lymphatic system, also leading to an unpredictable and premature antigen release. Advantageously, DCs in particular take up cationic NPs easily as compared to anionic and neutral carriers (Foged et al., 2005). Li et al. through polymerization reaction synthesized a copolymer named monomethoxy poly (ethylene glycol)-block-poly (2-(diisopropyl amino) ethyl methacrylate)- block-poly (2-(guanidyl) ethyl methacrylate); mPEG-b-PDPA-b-PGEM, PEDG. The nano self-assembly formed in an aqueous solution was reported to get activated in the cationic and acidic environment due to the presence of amino moieties causing a disassembled structure. PEDG nanoparticles represented to be an effective delivering agent by increasing the uptake efficiency of antigens by DCs (Li P. et al., 2017).

In terms of hydrophobicity, PLGA and chitosan have hydrophobic domains that activate the immune system due to their intrinsic adjuvant properties without the need of signal. This suggests hydrophobicity to be another important factor (Da Silva et al., 2009; Shima et al., 2013). The targeted delivery of adjuvants and antigens can be achieved by decorating the surfaces of NPs with specific ligands or antibodies, DNA, siRNA along with different dyes for image-guided combinatorial treatment (Norman Coleman, 1993; Roses and Czerniecki, 2014). In a study, Superparamagnetic iron oxide nanoparticles coupled with doxorubicin, indocyanine green imaging agent were camouflaged with cancer cell membranes providing simultaneous chemotherapy, hyperthermia and radiotherapy. The cancer cell membranes also preserved the surface antigens and other adherent molecules bestowing the system with good biocompatibility and tumor homing ability. The dual-modality imaging ensured the accumulation of the nanosystem in tumor and resulted good anti-cancer effects. It also reprogramed the macrophages from pro-tumor to anti-tumor (Huang Y. et al., 2018). Fluorescently labeled Poly (D, L-lactide-co-glycolide); PLGA NPs carrying toll-like receptor 7 (TLR-7) antagonist imiquimod (R837) in the core was encapsulated within cancer membranes and further reformed by mannose moiety (NP-R@M-M) to enhance uptake by antigen-presenting DCs (Yang et al., 2018). To improvise NP-R@M-M further for limiting tumor relapse, FDA approved PTT agent Indocyanine Green was conjugated, which upon exposure to NIR laser triggered tumor ablation and presented antigen in the form of dead cell fragments, thus helping to establish memory response (Chen Q. et al., 2016). Lipovaxin MM a liposomal formulation consisting multivalent DC-targeted allogenic vaccine for melanoma. Tumor antigens derived from plasma membrane vesicles were modified using a liposomal mixture with a metal chelating lipid 3 (nitrilotriacetic acid)-ditetradecylamine (3NTA-DTDA). A lipid envelope of α-palmitoyl-β-oleoyl-phosphatidylcholine (POPC) enabled superior insertion of the plasma membrane vesicles into the metal chelating lipid. The DMS-5000 DC targeting antibody fragments were then introduced in the metal chelating lipid through the poly-histidine C-terminal tail. The metal chelating linkage in the presence of nickel showed promising results in antigen expression (Gargett et al., 2018).

### Targeting Immune Checkpoint Agents

Naoparticles have evolved as delivery agents for immune checkpoint blockade and related activation mechanisms for cancer treatment. Apart from the effective delivery of drugs and vaccines, NPs shielding immune checkpoint inhibitors have been employed to boost immune response with minimized off-target effects. Inhibitors such as Cytotoxic T-lymphocyte associated antigen 4 (CTLA-4) and programmed death-ligand (PD-1/PD-L1) have been major points of focus in tumor immunotherapy due to clinical importance (Deng and Zhang, 2018). Classified in the family of B7 receptor CTLA-4 and PD-1 although have a synergistic inhibitory effect on T-cell activity; differences in distribution, time of action and signaling pathway mark their distinction (Fife and Bluestone, 2008; Keir et al., 2008). Hence, blockage of both CTLA-4 and PD-1 receptor-mediated pathways could result in the depletion of tumor growth through positive regulation of T-cell activity (Leach et al., 1996; Hirano et al., 2005). For example, NP systems like nanovesicles conjugated with PD-1 receptor when bound to tumor cells expressing PD-L1 ligand blocked the PD-1/PD-L1 axis stalling the growth of B16F10 Melanoma. As compared with free nanovesicles; PD-1 nanovesicles (PD-1 NVs) circulated for a longer time with a high accumulation rate in the tumor tissues. Also, the PD-1 NVs were able to reduce the growth of the tumor to a larger extent compared to anti-PD-L1 antibodies in mice with a higher survival rate (Zhang Xud. et al., 2018). Similarly in an investigation, NP based effective immune-checkpoint inhibitor delivery utilized poly (ethylene glycol)-block-poly (D, L-lactide); PEG-PLA nanoparticles comprising of CTLA-4 small interfering RNA (siRNA). Systemic administration of siRNA loaded PEG-PLGA reported an advent in the downregulation of Tregs and an elevation in CD4^+^ T-cells and CD8^+^ T-cells (Li et al., 2016). Another study was performed to improve the effect of PDT and deliver small interfering RNA (siRNA) to alter the PD-1/PD-L1 pathway. Blockage of the PD-1 receptor was achieved upon acidic cleavage of self-assembled pH-responsive poly (ethylene glycol)-block-poly (diisopropanol amino ethyl methacrylate-cohydroxyethyl methacrylate) micelleplex, covalently conjugated with Pheophorbide A (PPa) photosensitizer in hydrophobic core called as PDPA-PPa. PPa assisted multimodal imaging and PDT responsiveness. Cationic 1,2-epoxytetradecane alkylated oligoethyleneimine bonded high-affinity anionic siRNA co-self-assembled with PDPA-PPa formed a drug delivery system for Photodynamic cancer immunotherapy (Wang et al., 2016).

In recent work, Emami et al. demonstrated the regime of chemotherapy along with immune checkpoint inhibitors and PTT to increase therapeutic efficacy against cancer (Emami et al., 2019). Amide linkage after PEGylation of lipoic acid conjugated Doxorubicin a potent anticancer drug (LA-PEG-DOX) and anti-PD-L1 antibody (LA-PEG-PD-L1), assisted bond formation. This system was surface coupled with AuNP, a PTT agent through the dithiol covalent attachment and PEG-SH chain coated to stabilize the NPs. Upon NIR irradiation it was observed that chemo-photothermal immunotherapy targeting by PD-L1-AuNP-DOX nanoparticle formulation caused synergistic antitumor effect (Emami et al., 2019). Liposomal hybrid cerasomes containing porphyrin along with cetuximab (anti-EGFR antibody) in conjugation with a dye IRDye8s00CW and an MRI contrast agent DOTA-Gd, this combination of photodynamic therapy and PD-L1 have a synergistic anti-tumor effect, since they not only selectively targeted the tumor by effective accumulation at the site of action but also turned out as a promising strategy in preventing tumor relapse (Li Y. et al., 2018). One more experiment showed the possibility of combining the vaccine with immune checkpoint blockade therapy to retard tumor development (Chen Q. et al., 2016). A recent review comprehends information on emerging therapeutic strategies utilizing the molecular mechanism of reactive oxygen species (ROS) for inducing cancer cell death and apoptosis (Perillo et al., 2020). Oxaliplatin (OxPt), a cancer cell death-inducing agent and dihydroartemisinin (DHA) classified as ROS-producing drug respectively were loaded in core and shell of a nanoscale coordination polymer self-assembled NPs. DHA conjugation with the shell of NCP was supported by cholesterol linked disulfide bond masking it from systemic hydrolysis and reduction. Complete tumor elimination and prolonged memory response resulted when OxPt/DHA NPs along with anti-PD-L1 antibody was co-delivered to a mouse model. Also, the leftovers of cancer cellular content uptake by phagocytes caused activation of immune response thus improving the treatment efficacy (Duan et al., 2019).

## Preclinical and Clinical Outcome

After approval of the first liposomal formulation with drug doxorubicin, Doxil® by FDA and EMA, the market has seen the launch of many anticancer regimes (Barenholz, 2012; Leo et al., 2020). Sufficing the emerging need, the paradigm of approval has also come across inclination for theranostics including recently approved Novartis radiopharmaceuticals, Lutathera® ([^177^Lu]Lu-DOTA-TATE) for treatment of neuroendocrine tumor (Hennrich and Kopka, 2019) and many are being explored in the clinic (Table 2). Along with this, the combination of immunotherapy to widen the effect of nanomedicine for targeting other cells has been in demand to achieve higher order of tumor elimination (Tang et al., 2018; Duan et al., 2019).

**TABLE 2:** Recent clinical trials of cancer theranostic as reported by ClinicalTrials.gov.

S. No	Generic name	Target cancer	Status	ClinicalTrials.gov identifier ^a^
1	177Lu-PP-F11N	Medullary Thyroid Carcinoma	Phase 1/Recruiting	NCT02088645
2	18F-DCFPyL	Stage III ovarian cancer Stage IV ovarian cancer	NA/Recruiting	NCT03811899
3	177Lu-PSMA-617	Prostatic neoplasms	Phase 2/Recruiting	NCT04430192
4	90Y-DOTA-3-Tyr-Octreotide with 131I-MIBG	Neuroendocrine tumor	Early phase 1/Active, not recruiting	NCT03044977
5	111In-CP04	Medullary thyroid carcinoma	Phase1/Completed recruiting	NCT03246659
6	18F-FLT-TEP and 18F-FDG-TEP	Non-small cell lung cancer metastatic	Phase 2/Unknown recruiting status	NCT02069418
7	177Lu-PSMA617 with Cabazitaxel	Prostate metastatic cancer	Phase 2/Active, not recruiting	NCT03392428
8	68Ga-DOTA-JR11 and 177Lu-DOTA-JR11	Neuroendocrine tumors	NA/Active, not recruiting	NCT02609737
9	67Cu-SARTATE	Neuroblastoma	Phase 2/Recruiting	NCT04023331
10	Cu-64 SARTATE	Meningioma	Phase 2/Completed recruiting	NCT03936426
11	NG101m with temozolomide	Glioblastoma Multiforme	Phase 2/Not yet recruiting	NCT04373785

^a^Information collected from clinicaltrials.gov accessed on November 18, 2020.

The relationship between biomaterial and immunotheranostics has opened new investigations for clinical translations. Beyond the challenges of designing simpler nanoformulation incorporating complex multi-functional physicochemical properties of nanomaterial systems including size, shape, charge, composition and stability in preclinical assessment there are other hurdles (Lane et al., 2015). Reproducibility of data with accurate efficacy, scalability, standardization and filtration requires a thorough revision of protocol for improving outcomes in the clinical trial (Faria et al., 2018). Hence, adhering to patient compliance and stratification immunomodulatory nanoformulation should include screening based on acceptable therapeutic potential specifications (van der Meel et al., 2019).

Like all therapeutics, NP clearance has been of fidelity for conducting studies on liver and kidney as major eliminating organs even while assessing their accumulation in undesired areas (Longmire et al., 2008). Toxicity of nanomedicine is the most important criterion to be considered throughout *in vitro* and *in vivo* testing among which localized leads systemic delivery as localization produces minimum off-target effect due to focused cellular concentration (Linkov et al., 2008; Wang et al., 2015). More cautiously, systemic toxicity is prioritized when dealing with few antigens like lipopolysaccharides (LPS) (Shetab Boushehri and Lamprecht, 2019; Farhana and Khan, 2020) based on immune cell activation and delivery vehicles having inorganic NPs (Goodman et al., 2004; Maksoudian et al., 2020). Pharmaceutical formulations with biomaterial carrier for anticancer immunotheranostic application after clearance of toxicity test and related profiling which reports desired efficacy in the *in vivo* studies enters from pre-clinical into clinical phase (van der Meel et al., 2019). The traditional layout of cancer treatments reporting morbidity and mortality is not the only measurable result, but the recurrence rate analysis for rehabilitating healthy conditions has attracted equal importance while performing clinical studies (Chen J. et al., 2016).

## Conclusion and Outlook

In this review, we have tried to elaborate the interconnectivity between tailoring nanomaterial for various therapies. Nanoparticle as a single entity can be engineered to transport the therapeutically active agent for killing cancer cells aided by the immune response with real-time visualization tools like bioimaging. However, with diversification of heterogeneous cancerous disease state, treatment measures, and patient profiling the need for biomaterial qualifying for all-purpose has been inclined by a majority. Sophisticated and operational multimodality systems will eventually lead to the path of combining therapeutics and traversing the nanosystems through the complex biological *in vivo* environment with stability. Hybrid nanosystems demonstrating both therapeutic and diagnosis measures for cancer treatment have been highly influential and incline to expand exponentially.

Immunotherapy with its benefits has been favoring the future outcome due to its role in personalized medicines. Persistence will encompass drift toward synergizing nanomedicine and immunotherapy with relatively cheap, effective, and safer patient compliance than any monotherapy in cancer theranostic. One aspect to achieve this is building a nanobiomaterial system that acts as a carrier with diversified approach to regulate the immune system. The advent of biomaterial selection and design with multifunctional properties is highly recommended for demonstrating satisfactory carrier capability. Although, much of the research has been focused on launching a well-refined immunomodulatory biomaterial system but new emerging therapies and modification to existing strategies will invite revision of in-process protocols. Failure of many theranostics in clinical trial states the need to have better insight relating to immunotherapy and biomaterial for cancer cure. This could be due to the small subset of potential responses received by patients when using immunomodulatory nanohybrid systems. Biosafety and nanotoxicity of biomaterial are few important issues coming in the way of engineering structurally superior delivery systems. Rectification to all this can be achieved by a proper risk assessment of interaction and standardizing adaptable biomaterial to manipulate the immune response bridging the gaps of pre-clinical and clinical trials. A well-engineered biomaterial can even overcome tumor relapse and remission by bringing together the advantages of radiotherapy, chemotherapy, and immunotherapy which remains unexploited. Combined therapies with reduced off-target effects seem to have a much better evolving scope as compared to solely applied conventional methods thus, ushering the world into an advanced era for defeating oncology. Further, reducing immunotoxicity during and after systemic administration targeting tumor site captures more attention toward creating a novel concept in designing next-generation theranostic materials.

## Author Contributions

Conceptualization of manuscript, AK; Initial draft, AK, FD, SN, and BS; Final draft, AK; Review, BS, FD, and SN; Final edits, AK.

## Funding

This work was supported by the Department of Biotechnology, Government of India.

## Conflict of Interest

The authors declare that the research was conducted in the absence of any commercial or financial relationships that could be construed as a potential conflict of interest.
